# Gonorrhea in Women*An abstract of paper read at the Mississippi Valley Medical Association, Nashville, October, 1898.

**Published:** 1898-11

**Authors:** Joseph Rilus Eastman

**Affiliations:** Indianapolis


### GONORRHEA IN WOMEN.*

BY DR. JOSEPH RILUS EASTMAN, INDIANAPOLIS.

There is no malady to which womankind is liable that should engage more consideration than gonorrhea. Strewn in the train of its ubiquitous and wily germ are pathologic ravages of surpassing import in their clinical and moral consequences, yet hardly recognized or understood. Sinclair observed a decade ago strange indifference toward differential diagnosis of gonorrhea in the female, and even now, when in the light of recent bacteriology, it is easily possible to demonstrate the gonococcus in the act of pathologic mischief, there exists a notable, if not notorious, tendency on the part of most general practitioners, and some specialists, to disregard in doubtful cases discriminating diagnosis.

The diagnosis of acute gonorrhea in women is comparatively easy, even without the microscope. What with a history of impure coitus, free purulent secretion from vulva, vagina, and urethra, intertrigo, burning on micturition, and vesical tenesmus, the diagnosis is not far to seek. Upon inspection one usually detects a discharge of tenacious pus; or greenish or yellowish streaks upon the linen may alone be in evidence. Erosion may be present upon the skin, upon the lobia majora, or in the inguinal or gluteal folds. The labia minora, the clitoris and its prepuce and the hymen if present, are red and swollen. The meatus urinarius is found to be congested and ectropic, its normal pink color changed to a deep red. The mouths of the ducts of the Bartholinian vulvo-vaginal glands are deeper in color, gaping and tender. They discharge, instead of their normal glycerine-like secretion, pus laden mucous. A small area of mucosa immediately about these openings, exhibits a purplish red nuance. The surface of the vagina proper presents no great changes, the adult vaginal mucosa being practically uninfluenced by the presence of the gonococcus; not for the reason generally presented, namely, that the germ can not exist upon flat epithelium, but more probably because (as Doederlein has pointed out) of an acid environment and the presence of the vaginal bacillus.

It is certain beyond cavil that the gonococcus can grew fat upon flat epithelium. Rosinski reported five cases of gonorrhea of the mucous membrane of the mouth of the new born; and specific vaginitis in children is by no means rare. It occurs so often in plural instances among the children of the same household in Germany, that the term "gluckliche familie" has obtained general currency as a technical expression

*An abstract of paper read at the Mississippi Valley Medical Association, Nashville, October, 1898.

of the craft. The so-called "happy family" is one which father, mother and all of the children are simultaneously ill with clap.

A comprehensive examination of the discharge is not complete until the secretions of the urethra, Skene's lucunæ, the glands of Bartholini, the vagina and cervix, have been searched through; and in chronic cases several preparations should be made from each of these.

Before each act of removing discharge for examination, the vulva and the vestibule should be rinsed with warm, sterile water, and dried with cotton pledgets. The secretions of the urethral mucosa, this being the germ's favorite habitat, is generally sought first. The universal method of securing it is by milking the tract with the finger in the vagina, stroking it gently from the vesical opening to the orificum externum. If specific urethritis without discharge be present, nitrate of silver willgenerally bring the diplococci from the deeper layers. The urethra being emptied, any secretion present in Skene's glands may be evacuated by stroking with two fingers astraddle the urethra. The vulvo-vaginal glands will evacuate their discharge if compressed between the thumb and fore-finger.

To secure unmixed gonorrheal pus from the cervix uteri, care should be taken that the platinum wire does not come into contact with vagina or portic. It is best to first rinse, and then dry the vagina with cotton to free it of mucous.

It is not to be assumed that because the bacteria are intracellular that they are therefore gonococci; for it is certain that other diplococci than those of gonorrhea lie within the cell protoplasm. Moreover, the writer has seen preparations in which the unmistakable gonococci lay altogether without the cells.

The most distinguishing characteristic of the gonococcus as stained by the Pick method, according to Broese and Schillar, are the deep blue color, the biscuit or coffee-bean form, and their superior size. The gonococcus is an exceedingly large diploccoccus, averaging one and one-half micro-milimeters in length and three-fourths micro-milimeters in breadth.

It will be concluded after many examinations for gonococci that the urethra is the predilection seat of gonorrhea in women, and that the vulvitis and vaginitis are secondary, being caused by the bathing of these parts with the discharge from the urethra and cervix. Vaginitis and vulvitis are rarely seen in cleanly women, accoring to Bumm.

In many cases of endo-cervicitis, endo-metritis and salpingitis gonorrhea, it is impossible to isolate the specific germ.

A classical, clinical picture of gonorrhea is complete, and yet the most skillful attempts to run down the gonococcus are futile.

An adequate explanation of this paradox has been given us by Wasserman. He has shown that the gonococcus produces an active, specific poison. The poison is contained within the substance of the gonococcus itself. The poison remains active after the death of the germ. A very small amount of this poison if injected under the skin produces inflammation at the point of application, fever, swelling of the neighboring lymphatic glands and pain in the muscles and joints. Wasserman sought to find whether there is any immunity against this poison, with a negative result.

With these facts in mind, many dark places in the symptomatology of gonorrhea become more clear. It is possible to explain, by this token, blenorrhagic rheumatism after the gonococci have entirely disappeared. Moreover, the peculiar symptomatology of chronic gonorrheal inflammatian of the female genital apparatus becomes clearer.

Remembering that the gonococcus is ærobic and does not multiply under exclusion of oxygen, on the other hand very soon dies under this condition, the picture becomes still clearer. The cocci may become incapsulated and cut off from the circulation in an old exudate and die off, but at their death and disintegration the inflammation and fever-producing germs are set free. If they are absorbed, fever results; if they remain localized the local inflammatory process continues, perhaps indefinitely, since the organism can not immunize itself.

We have seen that the diagnosis of acute gonorrhea may be made by contemplation of the clinical phenomena alone; for example, if accute urethritis be present, we are almost certain that the gonococcus is to blame. A few day's observation will establish the diagnosis beyond conjecture, since the symptoms of non-specific urethritis will disappear rapidly. In chronic gonorrhea, however, too much dependence upon clinical manifestation is hazardous.

Condylomata are often present, but appear also often independently of gonorrhea. Debris-laden discharge from the vulvo-vaginal glands is usually an expression of old gonorrhea, but other germs, as the staphlococcus aureus and saprophitic forms, may occasion just such discharge. Among the more common indications of chronic gonorrhea are the maculæ gonorrhoicæ of Sanger, red papules about the openings of the vulvo-vaginal glands, sclerotic inflammation of these glands, in which the glands are felt as hard non-sensitive nodules under the examining finger, cysts of these glands and scars and erosions in the vulva. To these may be added the colpitis maculosa and granulosis.

All of these conditions may be caused by other germs than gonococci. Here, as in acute gonorrhea, the most reliable indication is the urethritis.—*Columbus Medical Journal.*

